# Evaluation of Antimicrobial Efficacy of UVC Radiation, Gaseous Ozone, and Liquid Chemicals Used for Disinfection of Silicone Dental Impression Materials

**DOI:** 10.3390/ma15072553

**Published:** 2022-03-31

**Authors:** Joanna Wezgowiec, Anna Wieczynska, Mieszko Wieckiewicz, Anna Czarny, Andrzej Malysa, Piotr Seweryn, Marek Zietek, Anna Paradowska-Stolarz

**Affiliations:** 1 Department of Experimental Dentistry, Wroclaw Medical University, 50-425 Wroclaw, Poland; joanna.wezgowiec@umw.edu.pl (J.W.); andrzej.malysa@umw.edu.pl (A.M.); piotr.krzysztof.seweryn@gmail.com (P.S.); marek.zietek@umw.edu.pl (M.Z.); 2 Department of Physicochemistry of Microorganisms, Institute of Genetics and Microbiology, University of Wroclaw, Przybyszewskiego 63/77, 51-148 Wroclaw, Poland; anna.wieczynska@pwr.edu.pl; 3 Department of Engineering and Technology of Chemical Processes, Faculty of Chemistry, Wroclaw University of Science and Technology, Wybrzeze Wyspianskiego 27, 50-370 Wroclaw, Poland; 4 Hirszfeld Institute of Immunology and Experimental Therapy, Polish Academy of Sciences, 12 R. Weigl St., 53-114 Wroclaw, Poland; czarnyanna7@gmail.com; 5 Division of Dentofacial Anomalies, Department of Maxillofacial Orthopedics and Orthodontics, Wroclaw Medical University, 50-425 Wroclaw, Poland

**Keywords:** dental materials, silicones, disinfection, ozone, UVC, chemical disinfection, oral pathogens, *Staphylococcus aureus*, *Pseudomonas aeruginosa*, *Candida albicans*

## Abstract

Effective disinfection of dental impressions is an indispensable requirement for the safety of dental personnel and patients. The ideal method should be not only effective but also convenient, cheap, and environmentally friendly. This study aimed to reliably evaluate the efficacy of ultraviolet C (UVC) radiation, gaseous ozone, and commercial liquid chemicals used for silicone dental impressions disinfection. These methods were applied to two types of elastomeric impression materials: condensation silicones and addition silicones of various consistency (putty, medium, and light). The antimicrobial effectiveness against *Pseudomonas aeruginosa*, *Staphylococcus aureus*, and *Candida albicans* was evaluated in vitro by counting colony-forming units (CFU) on the surface of samples. The one-way ANOVA with a Tukey HSD test or the Kruskal–Wallis with a Dunn’s test was performed. The results obtained revealed the efficacy of the proposed methods for disinfection of both C-silicones and A-silicones in most of the studied groups. Only one material (Panasil initial contact Light) was not effectively disinfected after UVC irradiation or ozone application. In conclusion, the potential of each disinfection method should be evaluated separately for each material. Moreover, in further research, the possible influence of the proposed methods on the physical properties of the impression materials should be thoroughly investigated.

## 1. Introduction

Dental impressions, which are used to create a negative form of the human dentition—teeth, hard and soft oral tissues—are a crucial prerequisite for the successful manufacturing of different types of oral appliances. Based on the dental impressions, three-dimensional replicas of intraoral situation are created in order to serve as working analogs used for the final work manufacturing [1]. A high-quality dental impression provides the technician with the possibility of fabricating a more precise dental prosthesis (e.g., crowns, bridges, partial or full dentures) or orthodontic splints and appliances, designed and manufactured individually to exactly match the oral conditions of each patient. Today, traditional physical impressions are increasingly replaced by digital intraoral scans, but scanners are still very expensive and less accessible for dentists, and therefore, conventional impressions are so far the method of choice in most dental offices [1]. Moreover, some reports revealed that the usefulness of scanners in highly demanding clinical situations with not spatially defined areas is still limited due to insufficient precision [2].

As dental impressions are taken intraorally by dentists and then must be transported to the dental laboratory technician, who further processes them to create positive reproduction of the teeth and other oral tissues, they must be considered as a potential source of infection in dental practice. This approach is justified by the fact that, on average, oral tissues are colonized with about 280 bacterial species, and 1 mL of a healthy person’s saliva contains approximately 750 million microorganisms [3]. For this reason, it is particularly important to properly disinfect all items that come into contact with the patient’s oral cavity to reduce the risk of transmission of pathogenic microorganisms. Careful carrying out of this procedure is necessary to effectively remove any microbial contamination present in the oral cavity, saliva, and blood and transferred into impression material [4]. At least 67% of dental materials received by dental laboratories, including dental impressions, were indicated to be contaminated by various microorganisms [5]. The most common microbes identified on the impressions are *Streptococcus* species, *Staphylococcus* species, *Escherichia coli* species, *Actinomyces* species, *Antitratus* species, *Pseudomonas* species, *Enterobacter* species, *Klebsiella pneumonia*, and *Candida* species [6,7].

Disinfection should be a routine procedure in dental offices and dental laboratories, allowing prevention of the spread of pathogens and limitation of biological risks of both self-contamination and cross-contamination at each stage of the fabrication of oral appliances, as impression, casts, and final works are transferred between dental office and laboratory several times [8]. In particular, the current situation of the SARS-CoV2 pandemic requires special care for safety concerns, highlighting the great importance of the effective disinfection of dental impressions [9,10]. Among the various available methods, autoclave sterilization would provide very effective microbial elimination, but, on the other hand, high temperature can cause damage to the material or loss of its properties, including tear and tensile strength, as well as dimensional changes [11]. Therefore, immersion and spraying are currently the most common techniques for disinfecting impression materials. The most popular agents used for this purpose are based on alcohols, aldehydes (glutaraldehyde 2%), chlorine solutions, phenols, biguanides, iodide combinations, and ammonium [12].

It must be taken into account that various disinfectants also pose a risk of alterations of dimensions and surface chemistry (e.g., wettability) of dental impressions. To avoid the negative influence of these chemical agents on different material properties of dental impressions, researchers are looking for other methods of disinfection, such as gaseous ozone [13], microwave irradiation [14,15], ultraviolet radiation [16,17,18], or electrolyzed oxidizing water [19,20]. Such alternative techniques may be particularly beneficial to oral health maintenance when applied to the most basic impression material used nowadays, based on sodium alginate. Alginate impressions are most commonly used in orthodontics or as the first impression in prosthetic and restorative dentistry. Due to hydrophilic properties, the use of spray or immersion for that material can cause impression distortion and deformations and result in dental cast distortion, affecting the accuracy of the final work [12,21]. For this reason, alginates, as well as polyethers, should not be immersed in disinfectants but only dipped quickly or sprayed. The other type of impression materials—silicones—are thought to be more resistant to the influence of external conditions. In particular, polyvinyl siloxanes (PVS, addition silicones) are characterized by high elastic recovery and are resistant to tearing and deformation. They are almost ideal elastic impression materials with 99.9% of elastic recovery (while for condensation silicones, it ranges between 98.2% and 99.6%) [22]. Demajo et al. demonstrated that silicone impression material exhibited a significantly lower microbial count than alginate material [23]. Moreover, the effect of immersion disinfection on dimensional changes of those types of impression material is rather low [24], although long-lasting disinfection (e.g., 18 h) is unfavorable and may significantly affect even such insusceptible impression material [25,26]. For these reasons, a detailed investigation of the potential of novel techniques of disinfection, other than spraying and immersion, is still highly recommended. In particular, UVC radiation and ozone, despite few previous research reporting usefulness of these methods for dental impressions disinfection, are still not gold standard techniques [13,16,17,18,19]. Apart from their application for surface disinfection, both these methods have the potential as a support for the treatment of endodontic infections and inflammation in root canals [27,28]. Hence, their effects on various types of dental silicones are an important, current scientific topic still requiring investigation.

This study aims to reliably evaluate the efficacy of ultraviolet C (UVC) radiation, gaseous ozone, and common chemical disinfectants (commercial spray and solution) applied to silicone dental impression materials. For this purpose, the effects of these methods of disinfection are compared for two types of elastomeric impression materials: condensation silicones (C-silicones) and addition silicones (A-silicones), contaminated with Gram-positive *Staphylococcus aureus* (*S. aureus*), Gram-negative *Pseudomonas aeruginosa* (*P. aeruginosa*), and the *Candida albicans* (*C. albicans*) fungus.

## 2. Materials and Methods

### 2.1. Sample Preparation

The dental impression materials selected for the study are listed in Table 1. They included two condensation silicones (C-silicones) and three addition silicones (A-silicones, Polyvinyl siloxanes) of different consistency (putty, medium, and light).

The preparation of the samples directly followed the manufacturer’s instructions and PN-EN ISO 4823:2015 [29]. After mixing, the materials were put into a metal mold to form disc-shaped samples with a 30 mm diameter and 6 mm height. Then, they were put in distilled water and placed in the incubator (CLN 15 Smart, POL-EKO-APARATURA, Wodzislaw Slaski, Poland) at 35 °C for the time defined by the manufacturer as the time in the oral cavity. After this conditioning, the samples were removed from the metal mold, rinsed with distilled water, air dried, and sterilized with UVC radiation (UV-C Blue, Activeshop, Wroclaw, Poland) for 50 min to avoid accidental contamination that could interfere with the microbiological test results.

### 2.2. Inoculation of Samples

After preparation, one surface of each sample was inoculated with a mixture of three microbial strains: *P. aeruginosa* ATCC 27853, *S. aureus* ATCC 6538, and *C. albicans* ATCC 10231 (ATCC, Manassas, VA, USA). The selection of strains was based on PN-EN 13727+A2:2015-12 [30] and PN-EN 13624:2013-12 [31].

The inocula were prepared to match the turbidity of the 0.5 McFarland standard dilutions (1.5 × 10^8^ CFU/mL) by transferring 1–2 colonies to the proper medium. A McFarland densitometer (DEN-1, BIOSAN, Jozefow, Poland) was used to control the density of the preculture and the final inoculum. Bacterial strains (*P. aeruginosa* and *S. aureus)* were cultured in Tryptic Soy Broth (TSB) medium (BTL, Lodz, Poland) at 35–37 °C for 18–24 h. *C. albicans* were grown on Sabouraud Dextrose Agar supplemented with 4% glucose (BTL, Lodz, Poland) at 28 °C for 48 h. The strains were then mixed, and the final inocula were prepared according to the ECS guidelines [32].

In the next step, impression material samples (*n* = 5 for each of 5 materials) were placed in separate Petri dishes and incubated in a mixed inoculum suspension at 37 °C for 30 min. Afterward, each sample was rinsed with 10 mL of water and subjected to the selected method of disinfection.

### 2.3. Disinfection of Samples

A description of four methods applied for disinfection of samples (UVC, gaseous ozone, commercial spray, and solution) is included in Table 2. 

Methods typically intended for dental impression disinfection (solution and spray) were used following the manufacturer’s instructions. UVC radiation was performed using an exposure time of 40 min, according to the recommendation of the UVC lamp manufacturer.

The ozone treatment was performed in a box with a volume of 8 L. First, ozone was generated for 9 min using an Ozox Professional G168 generator (MediaSklep24, Bojszowy, Poland) and pressed into the box to achieve a concentration of 15 ppm ozone. The samples were then placed in the box for 10 min under a further constant flow of ozone with a flow rate of 800 mg/h.

A positive, non-disinfected control was prepared for each of the studied materials.

### 2.4. Validation of the Method of Washing Away of Adhered Microorganism Cultures—MTT Assay

In order to validate the effectiveness of the method of washing away the adhered microorganisms from the surface of inoculated impression materials, the presence of microorganisms was assessed using the 3-(4,5-dimethylthiazol-2-yl)-2,5-diphenyltetrazolium bromide (MTT) metabolic assay, following the manufacturer’s protocol [33]. This assay is based on the ability of mitochondrial dehydrogenase in viable cells to cleave yellow MTT to violet formazan. After disinfection, the impressions were washed with water and placed in Petri dishes. Then, 200 μL of 5 mg/mL MTT (Sigma-Aldrich, St. Louis, MO, USA) solution was applied to each impression and incubated for 4 h at 37 °C. Subsequently, 200 μL of dimethyl sulfoxide (DMSO, Sigma-Aldrich, St. Louis, MO, USA) was added to each sample for 20 min to solubilize the formazan crystals. The amount of formazan dye produced correlates directly with the number of viable bacteria/fungi on the surface of the impression material.

### 2.5. Evaluation of the Efficacy of Disinfection—Microbial Cell Counting

To assess the efficacy of the selected disinfection methods, the residual microbial contamination of the impression materials was evaluated. After disinfection, the samples were rinsed with water, immersed in 10 mL of sterile phosphate-buffered saline (PBS, Hirszfeld Institute of Immunology and Experimental Therapy, Polish Academy of Sciences, Wroclaw, Poland), and vigorously shaken using IKA MS3 Basic Vortex (IKA, Staufen, Germany) for 3 min. Then, 1:10 and 1:100 serial dilutions of microorganisms in the PBS were plated on Mueller-Hinton 2 LAB-AGAR™ (MHA, BTL, Lodz, Poland) and Sabouraud Dextrose Agar (BTL, Lodz, Poland) and incubated at 37 °C for 24–48 h (for bacteria) or at 28 °C for 48–72 h (for *C. albicans*) in order to recover contaminating microorganisms. Finally, the plates were inspected for the presence of colony-forming units (CFU) of the selected strains. The data were transformed into a logarithmic scale in order to be presented as log_10_ CFU/mL.

### 2.6. Statistical Analysis

Statistical analysis of the obtained results was performed using GraphPad Prism 9.1.2. software (GraphPad Software, San Diego, CA, USA). All measurements were performed for the total *n* ≥ 15 for each group. The results were expressed as mean ± standard deviation. The parametric one-way ANOVA with a post hoc Tukey HSD test or the nonparametric Kruskal–Wallis with a post hoc Dunn’s test was applied to investigate differences between the studied methods of disinfection and non-disinfected control. Differences between groups were considered statistically significant with *p* < 0.05.

## 3. Results

**Figure 1:**
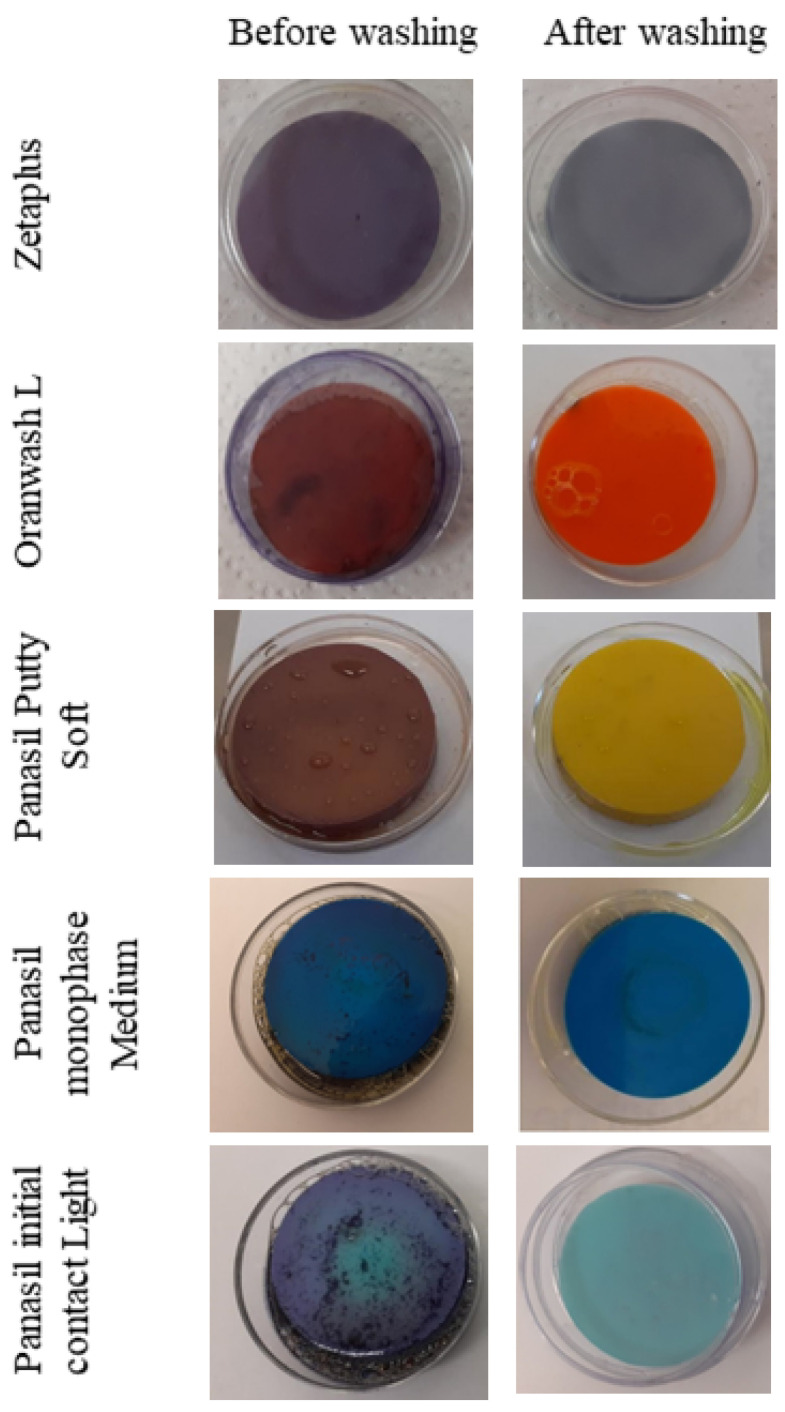
Validation of the effectiveness of washing away of cells adhered to the contaminated samples by immersion with PBS and vortexing. Before washing away, the presence of viable cells (a mixture of the oral pathogens: *S. aureus*, *P. aeruginosa*, and *C. albicans*) was demonstrated on the surface of all the dental impression materials studied. After washing, detachment of the cells was reached. *S. aureus—Staphylococcus aureus*; *P. aeruginosa—Pseudomonas aeruginosa*; *C. albicans—Candida albicans*; UVC—ultraviolet C.

The results of the MTT assay confirmed the effectiveness of the applied method of washing away cells adhered to the contaminated samples by immersion with PBS and vortexing (Figure 1).

Before washing away, the presence of viable cells was demonstrated on the surface of all the studied materials as a dark blue coloration. After immersion in PBS and vortexing, detachment of the cells previously adhered on the surface of dental materials was reached, enabling seeding of cells in the Petri dish and further processing of the experiments. The photos of the samples after washing present the original colors of the samples, confirming the lack of cells adhered to their surfaces (Figure 1).

**Figure 2:**
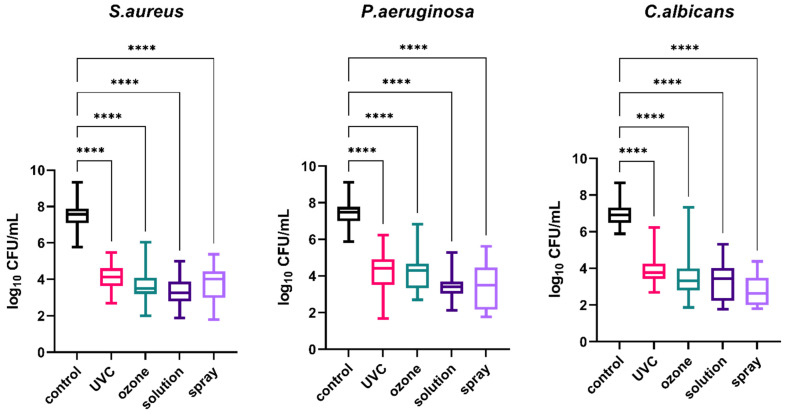
Effect of four methods (UVC, ozone, solution, and spray) used for the disinfection of all the studied materials (regardless of the type of material) contaminated with oral pathogens (*S. aureus*, *P. aeruginosa*, and *C. albicans*); the control material was contaminated but not disinfected; **** *p* < 0.0001 for all comparisons between the study (disinfected) groups and the control (non-disinfected) group. *S. aureus—Staphylococcus aureus*; *P. aeruginosa—Pseudomonas aeruginosa*; *C. albicans—Candida albicans*; UVC—ultraviolet C.

Comparison of the efficacy of different methods of disinfection (UVC, gaseous ozone, solution, and spray) applied to dental impression materials was the main objective of this study. To investigate the general effect of disinfection on impression materials regardless of the type of material, the results obtained for all materials were analyzed together. The results presented in Figure 2 demonstrate that microbial growth on the surfaces of materials contaminated with the pathogens studied (*S. aureus*, *P. aeruginosa*, and *C. albicans*) was significantly reduced after each type of disinfection compared to the non-disinfected control material (*p* < 0.0001).

**Figure 3:**
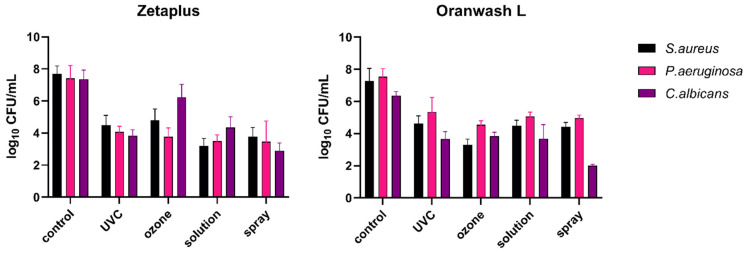
Effect of four methods (UVC, ozone, solution, and spray) used for the disinfection of C-silicones (Zetaplus and Oranwash L) contaminated with oral pathogens (*S. aureus*, *P. aeruginosa*, and *C. albicans*); the control material was contaminated but not disinfected; *p* < 0.05 for all comparisons between the study (disinfected) groups and the control group (non-disinfected); detailed results of statistical analysis are included in Table 3. *S. aureus—Staphylococcus aureus*; *P. aeruginosa—Pseudomonas aeruginosa*; *C. albicans—Candida albicans*; UVC—ultraviolet C.

**Figure 4:**
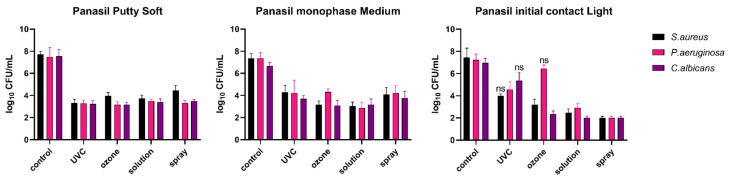
Effect of four methods (UVC, ozone, solution, and spray) used for disinfection of A-silicones (Panasil Putty Soft, Panasil monophase Medium, and Panasil initial contact Light) contaminated with oral pathogens (*S. aureus*, *P. aeruginosa*, and *C. albicans*); Control material was contaminated but not disinfected; *ns*—not significant (*p* > 0.05); for all other comparisons between the study (disinfected) groups and the control (non-disinfected) group, differences were significant (*p* < 0.05); detailed results of statistical analysis are included in Table 3. *S. aureus*—*Staphylococcus aureus*; *P. aeruginosa*—*Pseudomonas aeruginosa*; *C. albicans*—*Candida albicans*; UVC—ultraviolet C.

For a more detailed investigation, the analysis of microbial growth on the surfaces of particular dental impression materials (analyzed individually), disinfected with UVC radiation, gaseous ozone, Zeta 7 solution, and Zeta 7 spray after contamination with *S. aureus*, *P. aeruginosa*, and *C. albicans* was performed. The results obtained are presented in Figure 3 (for C-silicones: Zetaplus and Oranwash L) and in Figure 4 (for A-silicones: Panasil Putty Soft, Panasil monophase Medium, and Panasil initial contact Light). The summary of the results (means, medians, standard deviations) is also listed in Table A1. The results of the statistical analysis of the significance of the observed effects are listed in Table 3.

All disinfection methods evaluated were effective against selected oral pathogens (*S. aureus*, *P. aeruginosa*, and *C. albicans*) contaminating both types of C-silicones (Figure 3). For all of the studied groups, the applied methods of disinfection significantly reduced bacterial and fungal growth when compared to the non-disinfected control material (Table 3).

The comparison of the efficacy of different methods of disinfection of A-silicones is presented in Figure 4.

For Panasil Putty Soft and Panasil monophase Medium, similarly to the studied C-silicones, all evaluated disinfection methods were effective against selected oral pathogens (*S. aureus*, *P. aeruginosa*, and *C. albicans*) (Figure 4). For all of the studied groups, the applied methods of disinfection significantly reduced bacterial and fungal growth on the surface of these two materials when compared to the non-disinfected control material (Table 3). However, when Panasil initial contact Light was evaluated, low effectiveness of the selected methods of disinfection was revealed for the following conditions: UVC radiation against *S. aureus* (*p* = 0.9109), UVC radiation against *C. albicans* (*p* = 0.6131), and gaseous ozone against *P. aeruginosa* (*p* > 0.9999) (Table 3).

Summarized results revealing the efficacy of the proposed methods of both C-silicones and A-silicones disinfection in most of the studied groups are presented in Table 3. They are based on the statistical significance of the bacterial and fungal cell growth reduction observed compared to the colony-forming unit counted for the non-disinfected control samples. Only one material (Panasil initial contact Light) was not effectively disinfected using each of the studied methods. Differences between microbial growth observed in the non-disinfected control and in the samples subjected to UVC radiation were not statistically significant for *S. aureus* (*p* = 0.9109) and for *C. albicans* (*p* = 0.6131), while gaseous ozone was not effective against contamination by *P. aeruginosa* (*p* > 0.9999) of Panasil initial contact Light (Table 3).

## 4. Discussion

Development and validation of novel methods of disinfection for dental materials is a topic of high practical value, which could bring enormous benefits, both related to health and the economy. Although awareness of the necessity of the proper disinfection of dental impressions should not raise any doubts, this procedure is still neglected, in particular in developing countries [34]. Due to numerous weaknesses and limitations of the most popular techniques (immersion and spraying), in particular applied for hydrocolloid impressions, their modifications, including the incorporation of the antimicrobial agent to the impression material or mixing with antimicrobial solutions instead of water, have been proposed [35,36,37,38]. Furthermore, alternative methods of disinfection are still sought [39].

Although there are many studies focusing on the effectiveness of alginate impression disinfection, the number of reports concerning silicone disinfection is still limited. Taking into account the favorable properties of elastomeric impression materials and their growing popularity, particularly of polyvinyl siloxanes, these groups of materials deserve special attention in terms of the effect of various techniques of disinfection. To meet these expectations, our study assessed the potential of two methods, which are not commonly applied for disinfection of dental impression, i.e., UVC radiation and ozone treatment, for application to C-silicones and A-silicones.

Since UV chambers are commonly used for sterilization of dental instruments, they are available in most dental offices and laboratories. UVC irradiation may be used for microorganism inactivation via damage of the genetic material, which might cause malfunctions in cell replication [40]. Its potential for dental impression disinfection has been investigated by Aeran et al. It was confirmed that UV radiation significantly reduced the number of colonies of oral pathogens grown on the surface of all the studied materials used for taking impressions from the patients (alginate, addition silicone, and polyether). Moreover, the exposure time sufficient for total eradication of bacterial growth was 10 min for alginates, 15 min for A-silicones, and only 3 min for polyether. However, due to the fact that this study was conducted using impressions taken from the patients, there was no detailed information on the specific microbial strains present in the samples [16]. Another study demonstrated lower UV radiation effectiveness than autoclave, used for the elimination of pathogenic microorganisms from alginate impressions [17]. Godbole et al. revealed a lack of a significant negative influence of UV light disinfection on the dimensional stability of polyvinyl siloxane impressions. However, the antimicrobial effectiveness of UV light disinfection was not evaluated in this study [18].

As the existing evidence did not fully characterize the potential of using UV radiation for the elimination of pathogenic microorganisms from various dental impression materials, our results provide valuable insight into this problem. The study was carried out on two types of materials (addition and condensation silicones) of three different types of consistency, which covers a wide spectrum of modern impression materials used for various purposes. Our results confirmed the efficiency of UV light disinfection applied for four out of five tested materials. Disinfection of samples made of Panasil initial contact Light was not successful, which may result from different surface properties of this material, facilitating adhesion of pathogens and hindering its removal when compared to the other materials. Such an explanation could be supported by the results of the study of Giammanco et al., who confirmed that the efficacy of disinfection depends on the materials used for the impressions. One of the two commercial disinfectants studied (Sterigum) was less effective for the addition silicon (Elite) than polyether (Impregum) impression materials disinfection. On this basis, it was concluded that a need for immediate disinfection of impressions should be clearly indicated by manufacturers, and it must be strictly followed by the users [32].

Another low-cost method of disinfection is based on gaseous ozone, which is characterized by a high oxidizing capacity. This unstable molecule consisting of three atoms of oxygen can be used in dentistry for disinfection purposes because of its ability to inactivate bacteria, viruses, fungi, yeast, and protozoa. It can induce the oxidation of phospholipids and lipoproteins, leading to disruption of the integrity of the bacterial cell envelope [41]. The effectiveness of ozone in different virus types’ inactivation has also been confirmed [42]. In dentistry, one of the fields of using ozone includes removing the microorganisms from the oral cavity, as well as from dental unit waterlines and disinfection of dentures [43,44]. Fonseca et al. described the mechanisms of action of ozone as a powerful oxidant able to inactivate *Streptococcus mutans*, which is one of the principal etiological agents of carious lesions [45]. Although ozone has several advantages over other chemical disinfectants, such as lack of special requirements for storage or mixing, as well as lack of toxic residues harmful to the environment, it instead requires the purchase of a generator and proper, careful handling. Celebi et al. demonstrated a reduction of the growth of bacteria (*Escherichia coli*, *S. aureus*, *P. aeruginosa*, and *Enterococcus faecalis*) on the surface of A-silicone type impression material (Elite HD+; Zhermack) after treatment with gaseous ozone. Moreover, it had a positive effect on the wettability of the material [13]. Another study revealed that immersion of irreversible hydrocolloid materials in ozonated water can reduce the number of microorganisms (*S. aureus*, *P. aeruginosa*, and *C. albicans*) [43]. Similarly, our study also demonstrated the effectiveness of ozone treatment in the reduction of the growth of microorganisms on the surface of various elastomeric impression materials. The results obtained confirmed previous observations, indicating a need to evaluate each method separately for the specific material. It could be explained by the fact that due to the differences in the chemical composition and surface properties of various materials, the microbial adhesion and growth on their surface may differ. Indeed, it was revealed that the hydrophilic nature of alginates affects their susceptibility to contamination—alginate impressions are considered to have 2–5 fold higher microbial growth compared to elastomeric impression materials [46]. In light of this evidence, it should be concluded that the effectiveness of disinfection applied to various impression materials should be characterized thoroughly.

The limitations of this study are mainly related to the in vitro conditions. However, details of procedures performed in everyday clinical practice were carefully reproduced, and international standards were followed. Despite these facts, further research directions include a clinical study involving a real impression taken from the patient to take into account all possible microbial strains that are typically present in the oral cavity and contaminating dental impressions. Undoubtedly, the possible influence of the proposed methods on the physical properties of the impression materials also still requires detailed analysis, which will be a subject of our future study.

## 5. Conclusions

UVC radiation, gaseous ozone, and liquid chemical are similarly efficient and may be considered as silicone dental impressions disinfection procedures. For most of the studied materials, all of these methods enabled a significant reduction in the growth of common oral pathogens when compared to the non-disinfected control. However, since one type of A-silicone (Panasil initial contact Light) was not effectively disinfected, the potential of each method should be thoroughly investigated for each material separately. Moreover, the influence of UVC radiation and ozone on the physico-chemical and mechanical properties of silicone materials must be evaluated before recommending these low-cost and convenient methods as valuable alternatives of the classic techniques of dental impression disinfection.

## Figures and Tables

**Table 1:** Description of dental impression materials used in the study.

Type of Material	Consistency	Name	Manufacturer
C-silicone	Putty	Zetaplus	Zhermack (Badia Polesine, Italy)
	Light	Oranwash L	Zhermack (Badia Polesine, Italy)
A-silicone	Putty	Panasil Putty Soft	Kettenbach (Eschenburg, Germany)
	Medium	Panasil monophase Medium	Kettenbach (Eschenburg, Germany)
	Light	Panasil initial contact Light	Kettenbach (Eschenburg, Germany)

**Table 2:** Parameters of the disinfection methods applied in this study.

Method	Material or Equipment	Description
UVC	UV-C Blue (Activeshop, Wroclaw, Poland)	Irradiation for 40 min at 254 nm; power of 8 W, distance between the lamp and samples: 80 mm
Ozone	Ozox Professional G168 (MediaSklep24, Bojszowy, Poland)	Ozonation for 10 min at an ozone flow rate of 800 mg/h
Solution	Zeta 7 Solution (Zhermack, Badia Polesine, Italy)	Immersion for 10 min in 100× diluted solution and rinsing with distilled water
Spray	Zeta 7 spray (Zhermack, Badia Polesine, Italy)	Spraying and allowing to dry

UVC: ultraviolet C.

**Table 3:** Summary of the results of statistical analysis comparing the efficacy of different methods of disinfection of C-silicones and A-silicones; the table presents *p*-values from multiple comparisons test used to compare microbial growth after disinfection to the non-disinfected control; bolded *p*-values (>0.05) are considered non-significant, indicating lack of efficacy of the proposed method of disinfection.

Method of Disinfection	C-Silicones		A-Silicones		
	Zetaplus	Oranwash L	Panasil Putty Soft	Panasil Monophase Medium	Panasil Initial Contact Light
***S. aureus***					
**UVC**	<0.0001	<0.0001	<0.0001	<0.0001	**0.9109**
**Ozone**	<0.0001	<0.0001	<0.0001	<0.0001	0.0015
**Solution**	<0.0001	<0.0001	<0.0001	<0.0001	<0.0001
**Spray**	<0.0001	<0.0001	<0.0001	<0.0001	<0.0001
***P. aeruginosa***					
**UVC**	0.0060	0.0357	<0.0001	0.0024	0.0048
**Ozone**	0.0002	<0.0001	<0.0001	0.0007	**>0.9999**
**Solution**	<0.0001	0.0058	0.0149	<0.0001	<0.0001
**Spray**	<0.0001	0.0005	<0.0001	<0.0001	<0.0001
***C. albicans***					
**UVC**	<0.0001	0.0003	<0.0001	0.0030	**0.6131**
**Ozone**	<0.0001	0.0012	<0.0001	<0.0001	0.0002
**Solution**	<0.0001	0.0055	<0.0001	<0.0001	<0.0001
**Spray**	<0.0001	<0.0001	<0.0001	0.0458	<0.0001

*S. aureus*—*Staphylococcus aureus* (ATCC 6538); *P. aeruginosa*—*Pseudomonas aeruginosa* (ATCC 27853); *C. albicans*—*Candida albicans* (ATCC 10231); UVC—ultraviolet C.

## Data Availability

All data presented in this study are included in the published article or are available on reasonable request from the corresponding author.

## Appendix A

**Table A1:** Descriptive statistics for the examined samples.

Material	Pathogen	Method of Disinfection	Log_10_ CFU/mL		
			Mean	Median	SD
**Zetaplus**	*S. aureus*	Control	7.685	7.890	0.5063
		UVC	4.500	4.455	0.6063
		Ozone	4.808	4.780	0.6936
		Solution	3.207	3.000	0.4593
		Spray	3.782	3.890	0.5684
	*P. aeruginosa*	Control	7.412	7.120	0.8025
		UVC	4.068	4.085	0.3617
		Ozone	3.766	3.840	0.5546
		Solution	3.312	3.365	0.4501
		Spray	3.466	3.950	1.287
	*C. albicans*	Control	7.360	7.240	0.5651
		UVC	3.825	3.825	0.3826
		Ozone	6.240	6.105	0.7975
		Solution	4.343	4.420	0.6690
		Spray	2.905	2.980	0.4716
**Oranwash L**	*S. aureus*	Control	7.272	7.560	0.7835
		UVC	4.637	4.490	0.4694
		Ozone	3.295	3.300	0.3552
		Solution	4.487	4.480	0.3435
		Spray	4.428	4.430	0.2576
	*P. aeruginosa*	Control	7.535	7.650	0.5001
		UVC	5.342	5.760	0.9061
		Ozone	4.539	4.560	0.2567
		Solution	5.063	5.180	0.2687
		Spray	4.963	4.960	0.1899
	*C. albicans*	Control	6.356	6.380	0.2550
		UVC	3.658	3.560	0.4657
		Ozone	3.835	3.890	0.2446
		Solution	3.676	4.040	0.8783
		Spray	2.000	2.000	0.09126
**Panasil Putty Soft**	*S. aureus*	Control	7.724	7.710	0.2628
		UVC	3.329	3.330	0.3251
		Ozone	3.960	4.000	0.2952
		Solution	3.734	3.780	0.3058
		Spray	4.462	4.330	0.4559
	*P. aeruginosa*	Control	7.480	7.780	0.8451
		UVC	3.280	3.300	0.3065
		Ozone	3.156	3.220	0.2819
		Solution	3.496	3.500	0.1778
		Spray	3.312	3.360	0.2140
	*C. albicans*	Control	7.540	7.600	0.6089
		UVC	3.233	3.260	0.3088
		Ozone	3.156	3.130	0.2294
		Solution	3.416	3.430	0.2754
		Spray	3.492	3.480	0.1476
**Panasil monophase Medium**	*S. aureus*	Control	7.366	7.450	0.4230
		UVC	4.291	4.390	0.6401
		Ozone	3.162	3.190	0.3354
		Solution	3.048	3.180	0.3561
		Spray	4.075	3.945	0.6491
	*P. aeruginosa*	Control	7.363	7.480	0.5281
		UVC	4.207	4.670	1.170
		Ozone	4.318	4.355	0.2876
		Solution	2.865	2.850	0.5252
		Spray	4.229	4.000	0.6533
	*C. albicans*	Control	6.663	6.670	0.3241
		UVC	3.702	3.700	0.2846
		Ozone	3.096	3.060	0.4659
		Solution	3.183	3.300	0.5017
		Spray	3.775	3.880	0.6012
**Panasil initial contact Light**	*S. aureus*	Control	7.449	7.120	0.8477
		UVC	4.013	4.000	0.1653
		Ozone	3.189	3.350	0.4944
		Solution	2.460	2.360	0.3540
		Spray	2.000	2.000	0.1452
	*P. aeruginosa*	Control	7.227	7.340	0.5471
		UVC	4.574	4.840	0.6885
		Ozone	6.450	6.530	0.3190
		Solution	2.913	2.950	0.3807
		Spray	2.007	2.000	0.1448
	*C. albicans*	Control	6.966	7.000	0.4021
		UVC	5.356	5.530	0.7530
		Ozone	2.360	2.390	0.2690
		Solution	2.000	2.000	0.1594
		Spray	2.000	2.000	0.1355

CFU—colony forming unit; SD—standard deviation; *S. aureus*—*Staphylococcus aureus* ATCC 6538; *P. aeruginosa*—*Pseudomonas aeruginosa* ATCC 27853; *C. albicans*—*Candida albicans* ATCC 10231; UVC—ultraviolet C.
